# Impact of the RF Power on the Copper Nitride Films Deposited in a Pure Nitrogen Environment for Applications as Eco-Friendly Solar Absorber

**DOI:** 10.3390/ma16041508

**Published:** 2023-02-10

**Authors:** M. I. Rodríguez-Tapiador, J. Merino, T. Jawhari, A. L. Muñoz-Rosas, J. Bertomeu, S. Fernández

**Affiliations:** 1 Energy Department, CIEMAT, Av. Complutense 40, 28040 Madrid, Spain; 2 Technology Support Center CAT, University Rey Juan Carlos, Tulipán, s/n, 28039 Móstoles, Spain; 3 Unitat d’Espectroscòpia Raman, Centres Científics i Tecnològics de la Universitat de Barcelona—CCiTUB, Lluís Solé i Sabarís, 1-3, 08028 Barcelona, Spain; 4 Departament de Física Aplicada, Universitat de Barcelona, 08028 Barcelona, Spain

**Keywords:** copper nitride, reactive magnetron sputtering, novel absorbers, photovoltaic devices

## Abstract

This material can be considered to be an interesting eco-friendly choice to be used in the photovoltaic field. In this work, we present the fabrication of Cu_3_N thin films by reactive radio-frequency (RF) magnetron sputtering at room temperature, using nitrogen as the process gas. Different RF power values ranged from 25 to 200 W and gas pressures of 3.5 and 5 Pa were tested to determine their impact on the film properties. The morphology and structure were exhaustively examined by Atomic Force Microscopy (AFM), Fourier Transform Infrared (FTIR) and Raman Spectroscopies and X-ray Diffraction (XRD), respectively. The AFM micrographs revealed different morphologies depending on the total pressure used, and rougher surfaces when the films were deposited at the lowest pressure; whereas FTIR and Raman spectra exhibited the characteristics bands related to the Cu-N bonds of Cu_3_N. Such bands became narrower as the RF power increased. XRD patterns showed the (100) plane as the preferred orientation, that changed to (111) with the RF power, revealing a worsening in structural quality. Finally, the band gap energy was estimated from transmission spectra carried out with a Perkin Elmer 1050 spectrophotometer to evaluate the suitability of Cu_3_N as a light absorber. The values obtained demonstrated the capability of Cu_3_N for solar energy conversion applications, indicating a better film performance under the sputtering conditions 5.0 Pa and RF power values ranged from 50 to 100 W.

## 1. Introduction

Transition metal nitride materials such as copper nitride (Cu_3_N) show interesting outstanding properties, such as optical, electrical and energy storage properties, which have allowed this material to be used in several application fields. Specifically, Cu_3_N has attracted great interest as a new solar absorber material for flexible and lightweight thin film solar cells [1,2]. This metastable semiconductor is non-toxic, composed of earth-abundant elements and its band gap energy can be easily tunable depending on both the manufacturing conditions and the deposition methods. Among the application fields, it can be found in integrated circuits, photodetectors, optoelectronics, energy conversion applications and other technologies [3,4,5]. Highlighting the use of Cu_3_N, as a new solar absorber material for flexible and lightweight thin film solar cell technology [6]. Its development has attracted considerable attention with the purpose of being incorporated into novel designs within a future generation of cost-effective photovoltaic devices. This increased interest as an absorber in photovoltaic (PV) applications has mainly focused on its non-toxicity and its earth abundance, which makes it an eco-friendly material. In addition, the band gap energy value for the band gap of the Cu_3_N is ~0.9 eV [7,8], but the experimental results of the indirect and direct band gap show that they range from 1.17 to 1.69 eV and from 1.72 to 2.38 eV, respectively [8,9]. These obtained values mean that the Cu_3_N can be considered a potential next-generation solar absorber, a possible candidate to replace the silicon in solar cell applications. Such variations in the band gap can be attributed to a change in the stoichiometry of the Cu-N system and to the presence of unintentional oxygen impurities into the lattice. In addition, this material shows a very high absorption coefficient of 10^5^ cm^−1^ above ≈2.0 eV [10], which reinforces the idea of being used as an absorber for thin-film PV technologies [1]. Furthermore, this material can also show a p-type character, which can be obtained by doping it with interstitial fluorine [11]. The possibility to easily achieve both n and p characters by doping for the same material is useful. In this sense, the development of both p-type and n-type sandwiched thin layers, establishing an n-p junction to obtain power from sunlight in an economical way, entails an important saving in raw materials, among other advantages.

**Figure 1:**
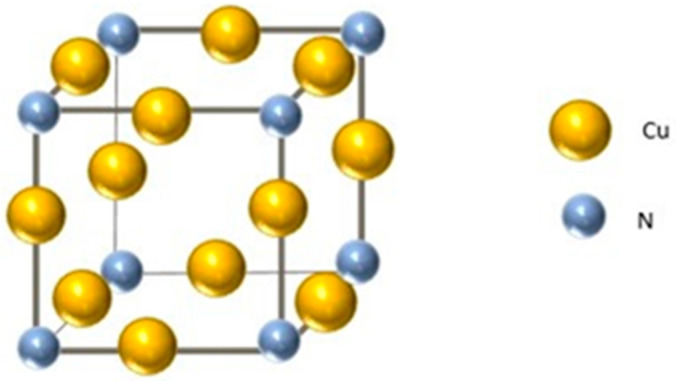
Picture of the anti-ReO_3_ crystal structure of the Cu_3_N compound. The copper atoms are represented by the yellow balls, and the nitrogen by the grey balls.

At room temperature (RT), Cu_3_N is a metastable semiconductor with an anti-ReO_3_ cubic crystal structure (see Figure 1). Its structure exhibits the nitrogen (N) atoms at the corners of the unit cell and the copper (Cu) atoms, located at the center of the cubic edges. Because of that, this crystal structure is extremely favorable to insert metal atoms at the interstitial body center site (½,½,½) [12], a fact that could lead to different chemical interactions between Cu and N atoms, affecting the electronic structure of the material. Among the most common elements that can be used to be inserted in that position, we can find alkali metals (Li) [13], transition metals (Ti, Pb, Ni, Zn, Cr, Fe, Mn, Al and Sc) [14,15,16] and/or non-metals (H and O) [17,18].

Depending on its chemical composition, the Cu-N compound family can offer a wide variety of optoelectronic properties, i.e., the Cu-rich Cu_4_N films that shows a metallic behavior, the Cu-rich, N-rich and stoichiometric Cu_3_N films that present a semiconducting performance [19]. Hence, it is clear that there is a strong relationship, between its chemical bonds and its electronic properties. This fact is mainly attributed to the hybridization effect between the Cu 3d-N 2p bands and the Cu (4s, 4p) conduction bands, which gives rise to the desired covalent bonding effect [7].

On the other hand, the different natures observed in this material can be reached by modifying its deposition conditions and/or depending on the fabrication technique used. The differences in morphological, structural, chemical and optoelectronic properties reported in the literature are attributed to the vacant interstitial sites occupied by atoms other than Cu and the chemical interactions between them [20,21,22]. That is why the knowledge of its local atomic structure is of great importance; it permits the determination of the most suitable material that fits the device requirements. In addition, by modifying the deposition conditions, both the lattice constant and the band gap energies can also be easily tuned, from 0.3815 to 0.3885 nm [23], and in a surprisingly wide range of values from 0.23 to 1.90 eV [24], respectively. Many reports have shown that the abovementioned material has been fabricated by different approaches, such as pulsed laser deposition (RPLD) [25], molecular beam epitaxy (MBE) [26], atomic layer deposition (ALD) [27], ion-assisted deposition [26], direct current (DC) triode sputtering [28] and magnetron sputtering [29]. Among them, one of the most commonly used approaches is reactive radio-frequency (RF) magnetron sputtering due to the ease with which this technique modifies the film properties by changing the deposition parameters. It is also preferred because of its great capability of scalability to large areas [4]; it is a reliable, robust and cost-effective technique that can be easily incorporated into an industrial production chain. In this sense, Cu_3_N films with different structural, optoelectronic properties have been already reported by modifying parameters such as the nitrogen fraction in the gas mixture, the power value, the type of substrate, the substrate-to-target distance and the substrate temperature, among others [23,29,30,31].

Finally, the thermal decomposition of Cu_3_N is a parameter that should also be considered because it occurs at a temperature that is not relatively high, from 100 to 470 °C. Okrasa et al. [32] reported that, at a high temperature of 200 °C, structural changes related to the release of pure Cu could be observed.

This work presents the fabrication of a Cu_3_N binary compound by reactive RF magnetron sputtering at RT in a pure N_2_ environment by modifying the RF power and the total working pressure. In our previous works, we observed that the use of an argon (Ar)-free environment during the sputtering process led to films with better structural quality, the (100) plane being the preferential orientation, and smoother surfaces when Ar was used during the sputtering process [33]. Considering that the design of desired functional material properties by controlling deposition parameters is a key technological topic, in this work, we establish the crystal nature, morphology, electrical and optical properties of the deposited Cu_3_N films as functions of the preparation conditions. Our main goal is to study the impact of both the RF power and the gas pressure on the thin film properties, and to then adjust them according to the device requirements. Thanks to the possibility of such tuning, an ad hoc material could be fabricated with suitable properties to be used as a potential substitute absorber of silicon in PV devices. Finally, the sputtering conditions that lead to a best thin film performance are discussed.

## 2. Materials and Methods

The deposition of the Cu_3_N films was carried out using a commercial MVSystem LLC (Golden, CO, USA) mono-chamber sputtering system, where the gun was radio-frequency (RF) operated and vertically adjustable. The substrates used were Corning glass 1737F (Corning Inc., New York, NY, USA) and <100> polished n-type floating zone crystalline silicon (c-Si) wafers. The 3-inch diameter and 6-mm-thick Cu target was from Lesker Company (St. Leonards-on-Sea, East Sussex, UK) and had a purity of 99.99%. Prior to loading the substrates into the sputtering chamber, native silicon dioxide was removed from the surface silicon wafer with a 1% HF solution in deionized water and ethanol for 5 min. On the other hand, the glass was ultrasonically cleaned for 10 min with ethanol and deionized water, and it was finally immersed in isopropyl alcohol. Afterwards, all substrates were dried by blowing nitrogen over them. The sputtering chamber was pumped to a base pressure of 10^−5^ Pa, whereas the sputtering process was carried out in an Ar-free environment with the total working pressure set to 3.5 and 5 Pa, respectively, adjusted with a “butterfly” valve. The process gas was nitrogen (N_2_) (99.999%), with a flow rate of 20 sccm controlled by a mass flow controller (MFC) (MKS Instruments, Andover, MA, USA). The distance between the target and the substrate was set to 10 cm. The RF power varied from 25 to 200 W, and the deposition time was modified from 420 to 1800 s to offset the effect of the power. In all of the experiments, the substrate was not intentionally heated.

The thickness, the structure, the morphology and the chemical composition of the Cu_3_N films were studied by profilometry, X-ray diffraction (XRD), atomic force microscopy (AFM), Energy-Dispersive X-ray Spectroscopy (EDS), Raman and Fourier transform infrared spectroscopy (FT-IR) in transmission mode, respectively. The thickness was measured using a Veeco Dektak 8 Stylus profiler. The crystal structure, the grain size and the lattice parameters were determined from the XRD diffraction pattern, measured with a *Panalytical* powder diffractometer, model X’Pert MPD/MRD, using the Cu-kα radiation (λ = 0.15406 nm). The scanned 2θ range was 10–60° at a step size of 0.1°. The topography of the Cu_3_N was evaluated with a multimode nanoscope AFM model III A (SPM, Veeco-Digital instrument) in tapping mode, using silicon nitride AFM tips (OTR8, Veeco). The roughness of the samples was quantified by root mean square (RMS); meanwhile, the grain size was also determined, in both 1 × 1 μm^2^ 2D AFM images using a commercial software (Gwyddion software 2.61, http://gwyddion.net/) (accessed on 31 January 2023). A semi-quantitative elemental chemical analysis was carried out using an Oxford detector energy dispersive X-ray spectroscopy (EDS) system equipped with a high-resolution field emission SEM (JEOL JSM 6335F). Determinations of the molecular structure were performed with a dispersive spectrometer Horiba Jobin-Yvon LabRam HR 800 coupled to an optical microscope Olympus BXFM, using a solid-state laser as an excitation source emitting at 532 nm. Furthermore, the laser power at the sample was around 5 mW and the used microscope objectives were 100× and 50×. A Perkin Elmer Spectrum 100 FT-IR was also used to complement the information obtained from the Raman measurements. The spectra were measured in the transmittance mode in the wave number, which ranged between 400–4000 cm^−1^. Electrical properties, such as sheet resistance and resistivity, were obtained with a commercial 4-point probe measurement system (Signatone, EEUU). It was estimated that the errors in the measured parameters were around 2%. Finally, the optical transmittance spectra were measured at normal incidence using a UV/VIS/NIR *Perkin Elmer* Lambda 1050 spectrophotometer. From these spectra, the optical band gap energies E_g_ from the indirect and direct transitions were calculated to determine the most favourable sputtering conditions, which obtained a suitable solar absorber. The glass substrate was used to characterize the films by profilometry, XRD, AFM and optical properties. On the other hand, the silicon substrate was used to carry out the Raman and FTIR measurements.

## 3. Results and Discussion

All of the Cu_3_N films presented in this work were physically stable with good adherence to the substrates after exposure to ambient air. No cracking or peel-off was observed after the deposition. Table 1 describes the sputtering deposition conditions used and the measured film thickness of the samples.

**Figure 2:**
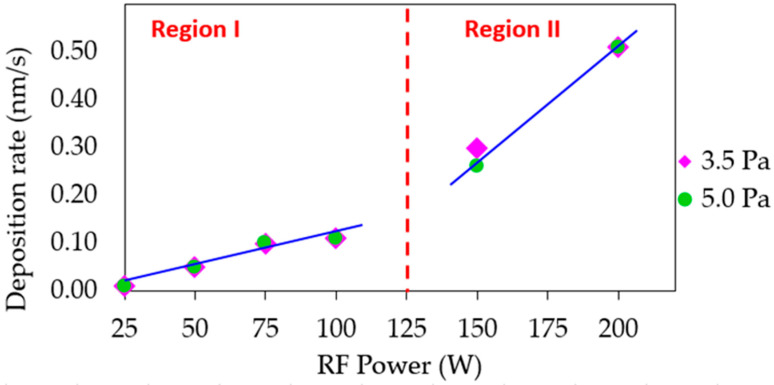
Deposition rate calculated for Cu_3_N samples prepared at different RF power values and gas pressures. The line is a visual guide.

Figure 2 shows the deposition rate as a function of the RF power values used. This parameter was calculated from the Cu_3_N film thickness, data obtained with the profilometer and the deposition time (see data in Table 1). In spite of trying to adjust the deposition time to obtain similar thicknesses, film thicknesses ranged from 40 to 215 nm. As expected, the deposition rate increased with the RF power. This tendency can be attributed to the enhancement of the Cu atoms flow obtained as RF power rises at a constant gas pressure. It should be pointed out that a linear trend with two different slopes was observed as a function of the RF power value. This indicates that two different growth regimes were taking place, which had an effect on the film properties. On the other hand, there was almost no difference between the rates measured at the two different gas pressures used in this work. This would mean that the modification of the plasma species due to the increased gas pressure was not great enough to lead to a higher number of collisions between them. Therefore, at a higher pressure of 5.0 Pa, the sputtered Cu atoms still reached the substrate surface by losing a similar amount of energy as that lost at the lower pressure of 3.5 Pa due to no meaningful increase in collisions taking place.

**Figure 3:**
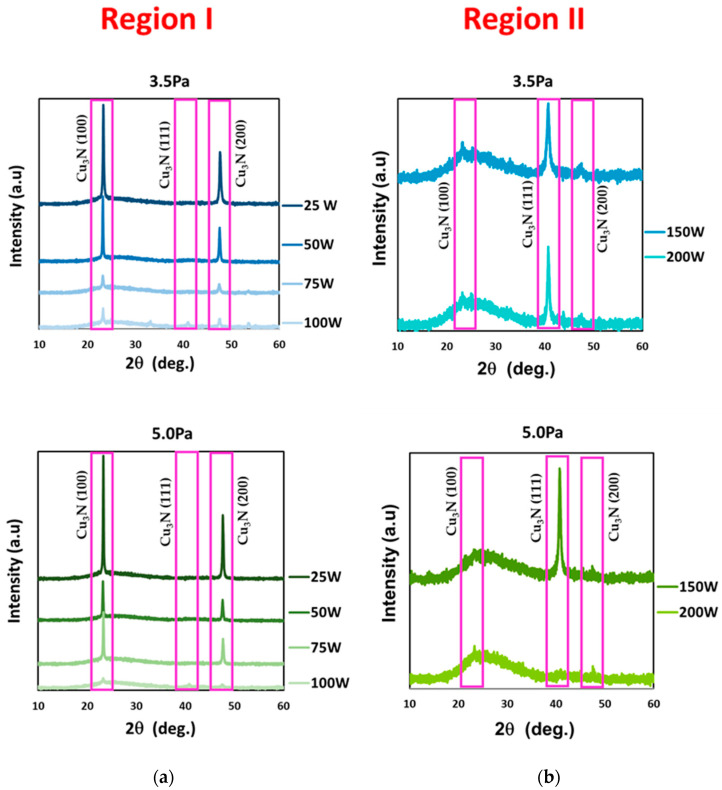
X-ray diffraction spectra of Cu_3_N films deposited at gas pressures of 3.5 Pa and 5.0 Pa and the different RF power of (**a**) 25 W to 100 W (region I) and (**b**) 150 W and 250 W (region II).

Figure 3 shows the XRD patterns of the Cu_3_N films deposited on glass at the N_2_ gas pressures of 3.5 and 5.0 Pa, and the RF power values of 25 W to 100 W (region I) (Figure 3a) and 150 and 200 W (region II) (Figure 3b). All of them revealed a polycrystalline nature with an anti-ReO_3_ structure, typical of the cubic Cu_3_N (card n° 00-047-1088), and no evidence for Cu-phase and CuO formation were observed. A predominant (100) direction was obtained at RF power values up to 150 W. This preferential orientation, referring to the (100) and (200) diffraction planes, are related to an N-rich growth (Figure 3a) [34]. This N-rich growth mode was attributed to the low amount of Cu atoms within the plasma at such values of RF power. As the RF power increased, a transition to the dominant (111) plane was observed, which corresponded to a change in the growth mode towards a Cu-rich material (Figure 3b) [35,36,37]. The beginning of an amorphous matrix was observed in samples from region II deposited at different pressures at RF powers of 150 and 200 W, being more evident in the case of the sample at 200 W and 5.0 Pa, (see Figure 3b), where the (100), (111) and (200) diffraction planes could be intuited, with the (100) plane being the most intense. In comparison with the sample deposited at 200 W and 3.5 Pa, which clearly presented the dominant (111) plane, the amorphous nature of that sample could be attributed to a saturation effect of the Cu_3_N film reported by other authors [38], which may take place when the gas pressure rises above a certain value. Taking the above results into account, the Cu_3_N films presented a possible over-stoichiometry that would mean additional N atoms inserted into the lattice, probably as interstitials. This stage would be more evident as the gas pressure increased, due to an enhancement in the number of energetic and ionized N species within the plasma. On the other hand, the (100)-oriented material seemed to be preferred to the (111)-oriented material. As Z. Cao et al. previously reported [39], the Cu_3_N material with the (111) plane as its preferred orientation showed, at the grain boundaries, Cu atoms agglomerated, forming nanocrystals. Hence, these samples could give rise to an uncertain factor in determining their optical and electrical properties. In fact, Y. Du et al. [40] also observed a sudden drop in the measured electrical resistivity of the (111)-oriented Cu_3_N material, due to an additional conductance mechanism, causing the percolation effect to come into play, which was not beneficial when using such oriented material as a solar absorber. Finally, it should be pointed out that the number of diffraction peaks that appeared in the patterns of the samples fabricated in the N_2_ pure environment (see Figure 3) were less than those obtained in our previous work for the samples fabricated in a mixture of Ar and N_2_ [33]. This fact could positively contribute to minimizing trapping centers due to the reduction of grain-orientation effects, being indicative of a better crystal quality.

As the information of the local atomic structure is very important, the lattice constant a was calculated from the data obtained in the XRD patterns, using the following Equation [41]:

(1) $$d\left(hkl\right)=\frac{a}{\sqrt{\left(h^{2}+k^{2}+l^{2}\right)}}\;\;\;$$

where  h, k and l are the Miller indices. The lattice parameter refers to a stoichiometric and stress-free sample, which is 0.38170 nm and 0.38192 nm for the (100) and (111) planes, respectively. The grain size (τ) was also determined using the *Debye-Scherrer* Equation (2) [42], where k is a constant (0.9), λ is the X-ray wavelength (0.154 nm), θ is the diffraction angle and β  is the full width at half maximum (FWHM) of the predominant peak.

(2) $$\tau =\frac{k\lambda }{\beta \cdot \cos\theta_{B}}$$

The FWHM, the grain size and the lattice parameters are summarized in Table 2 as functions of the RF power, referring to the predominant (100) and (111) planes in each case.

According to data in Table 2, the (100) diffraction peak shifted to lower values of angle 2θ (reference position 2θ = 23.285°) when RF power increased. On the other hand, the lattice parameters calculated were slightly higher than the theoretical parameters (0.38170 nm). This demonstrates the over-stoichiometry of the film in terms of N content, as other authors have previously reported [20,37,43]. With regard to the mean grain size calculated, no clear trend was observed in RF power for the different sample series of gas pressures, with the values being around 27–38 nm, similar to those obtained in the literature [9,32,37]. A higher grain size was observed, as highlighted for the samples in region I; hence, a better crystal quality was expected, as confirmed in the following paragraph by the analysis of the FWHM values of the dominant peak.

**Figure 4:**
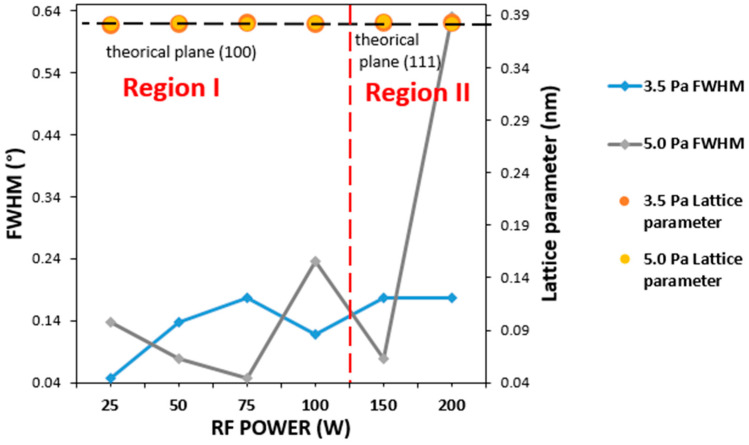
FWHM and lattice parameters extracted from the XRD spectra of Cu_3_N films deposited at the gas pressures of 3.5 Pa and 5.0 Pa and the different RF powers of 25 W to 100 W (region I) and 150 W and 250 W (region II).

Figure 4 shows the evolution of the FWHM and the lattice parameters as functions of the RF power. Two different regions can again be defined: region I, corresponding to the samples that showed the (100) plane as the preferred orientation, and region II, represented by the samples with the (111) plane as the preferred orientation. In region I, the samples deposited at 3.5 Pa presented a minimum FWHM value of 25 W, whereas this minimum was shifted to 75 W in those deposited at 5.0 Pa, indicative of improved structural quality. This displacement of the RF power value at which the minimum is reached could be explained by the slight difference in the number of N species within the plasma at the working gas pressures used. As the RF power increased, a high number of Cu atoms with high energy and enhanced mobility reached the surface, resulting in improved crystallinity. However, in the case of 3.5 Pa of gas pressure, fewer N atoms would be in the plasma while the number of Cu atoms increased with the RF power; hence, there may be an excess of the latter. In addition, the probability of the formation of defects due to disorder at the Cu and N sites increased. This is what occurred at 3.5 Pa and RF powers above 25 W, where a detriment in the crystal quality was observed as RF power increased. In contrast, at 5.0 Pa, the excess of Cu atoms began to be compensated by the increase of N species within the plasma to form the bond Cu-N. For this reason, the sample with improved crystallinity was deposited at a higher RF power value of 75 W. On the other hand, in region II, no clear trend was observed for the samples deposited at 3.5 Pa; but a huge increase in FWHM was obtained for the sample deposited at 5.0 Pa and 200 W, attributed to its amorphous character.

**Figure 5:**
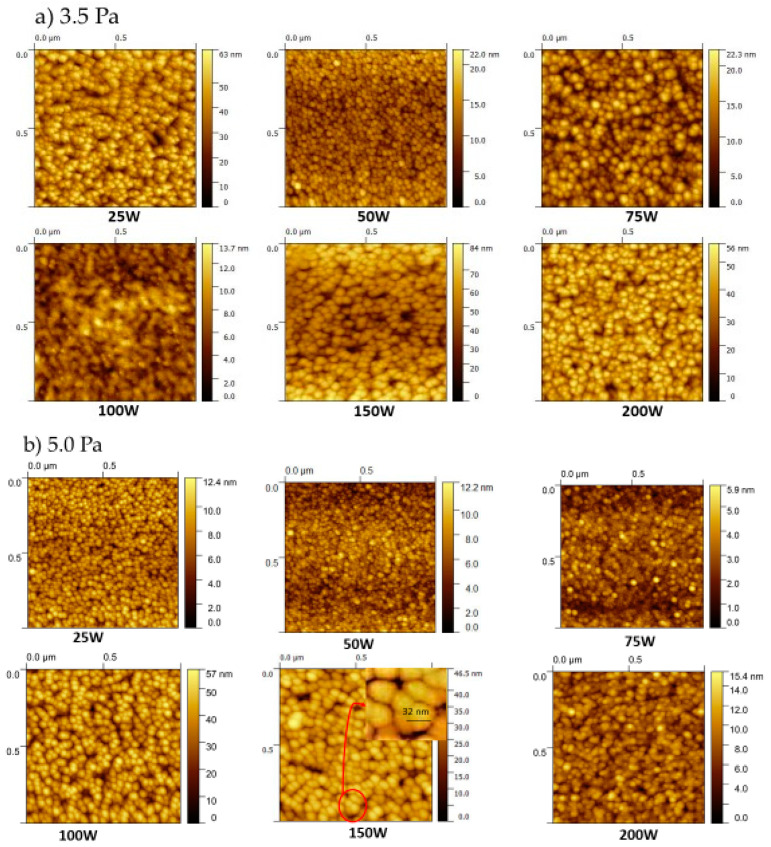

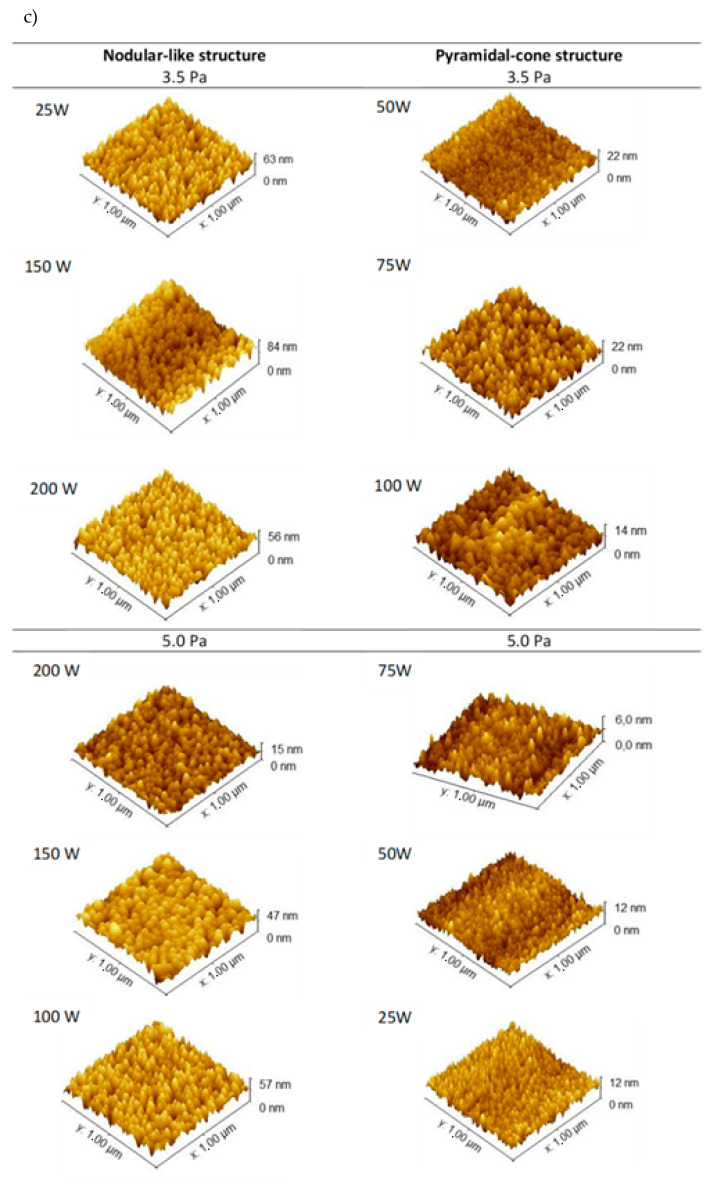
1 × 1 µm^2^ 2D AFM micrographs of the sputtered Cu_3_N films deposited at different RF powers and at (**a**) 3.5 Pa and (**b**) 5.0 Pa; (**c**) 1 × 1 µm^2^ 3D AFM micrographs divided depending on the type of morphology.

The surface morphology of the samples was studied by AFM. Figure 5 depicts the 1 × 1 µm^2^ 2D micrographs of the sputtered Cu_3_N films at 3.5 Pa, in Figure 5a, and 5.0 Pa, in Figure 5b. Table 3 summarizes the grain size and the surface roughness of the Cu_3_N films calculated with a commercial software (Gwyddion software) by using the 1 × 1 µm^2^ 2D AFM images. These calculations led to an estimated error of 10%. According to the results obtained, all of the films presented smooth surfaces, with a grain size that was not strongly influenced by both the RF power and the N_2_ gas pressure used. However, it was observed that the grain shapes were slightly different depending on the sputtering conditions used. In this sense, two types of morphologies were achieved in agreement with the two regions defined from the XRD results: pyramidal-cone and nodular-like structures, attributed to both the transition observed in the crystalline orientation and the increase of the deposition rate due to the rise of the RF power (see Figure 2) [44]. The films of region I prepared at 3.5 Pa of N_2_ gas pressure showed a conical morphology attributed to the strong (100) preferred orientation [45]. Large polygonal grains with irregular shapes were separated by many voids. As the RF power increased, the grains began to be agglomerated to form a big grain, a fact attributed to their higher number of grain orientations; see Figure 3a. This agreed with the worsening observed in the crystallinity of such films by XRD, where, in region I, as the intensity of the (100) diffraction peak decreased with increasing RF potential, the (111) peak appeared more intense. On the other hand, the morphology of the samples in region II, deposited at 150 and 200 W showing the (111) plane as the preferred orientation, showed nodular-like structures [46]. In the case of the samples prepared at 5.0 Pa, a similar trend was observed.

Grain size ranged from 29 to 37 nm and was estimated from the AFM 2D images (data in Table 3), close to the values obtained from the XRD measurements by using the Debye-Scherrer Equation (2). These data revealed similar grain sizes regarding the sputtering conditions. However, a different grain morphology was also observed in the (111)-oriented films. Such samples presented small agglomerated grains that led to visibly larger grains, as the corresponding AFM micropraphs of Figure 5 showed. This effect of the agglomeration of grains may be attributed to nitrogen loss, which is more favorable in the (111) planes. This orientation was obtained as the RF power increased (samples deposited at 150 and 200 W), as shown in the XRD patterns (Figure 3). Finally, a slight reduction in surface roughness RMS by increasing the gas pressure was observed at low RF power values. As the working pressure increased, more N_2_^+^ ions struck the Cu target, leading to a higher energy of the ion bombardment on the substrate, and hence, more atom diffusion. In this scenario, the density of the films was enhanced and the surfaces were flattened [47]. In addition, the samples deposited at 150 and 200 W (samples in region II) presented higher RMS values than the other samples, which is attributed to the observed changes in morphology and in the preferred orientation, as previously explained. The highest RMS values were shown by the samples deposited at 25 W and 3.5 Pa, and 100 W and 5.0 Pa, which were attributed to the presence of many deep voids on their surfaces. Therefore, the sputtering conditions that led to the smoothest surfaces together with an improved crystallinity were an RF power of 75 W and a gas pressure of 5.0 Pa.

Chemical composition was qualitatively determined from EDS data, as summarized in Table 4. These data revealed a qualitative ratio of Cu to N lower than 3, confirming the non-stoichiometry of the fabricated material [48,49]. From these measurements, the samples showed a slight increase in the Cu/N ratio as the RF increased due to the increase in the amount of Cu atoms; but no significant increase of N in the network was observed with rising gas pressure, as we argued when determining the deposition rate. The samples deposited at 200 W showed the highest presence of Cu in the lattice, as expected.

**Figure 6:**
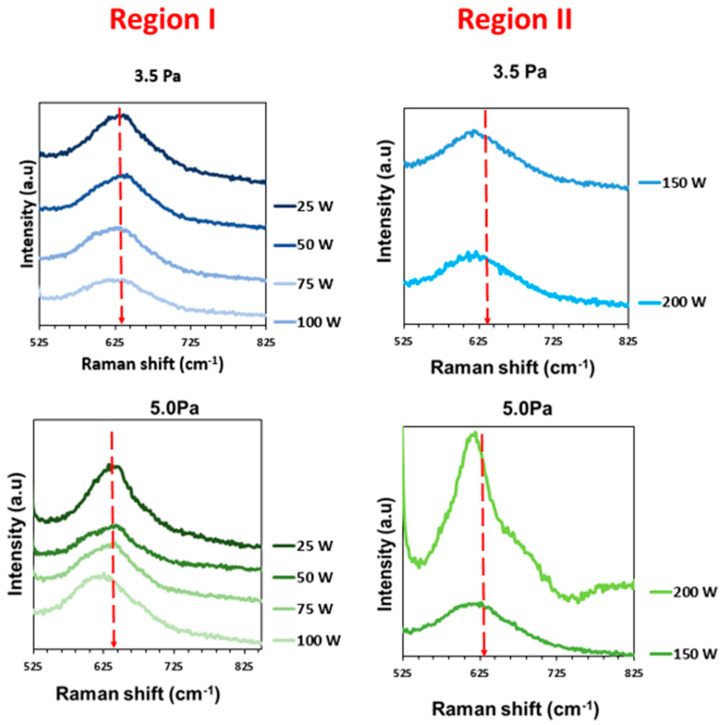
The Raman spectra of Cu_3_N films prepared at different RF powers and gas pressures of 3.5 Pa and 5.0 Pa. (25–100 W, region I, and 150–200 W, region II).

Figure 6 shows the prominent Raman peak at around 613–644 cm^−1^, characteristic of the formation of the Cu_3_N, and which corresponded to the stretching of the Cu–N bond [50]. A Raman shift at a lower frequency was observed as the RF power rose, as well as when increasing the gas pressure. This shift would imply a deviation from the stoichiometric level, as already previously observed. That is, at a high RF power, the presence of Cu in the lattice is more evident. In addition, the shape of the main peak was asymmetric, which was more evident for samples in region II.

Table 5 summarizes the main parameters derived from Raman spectra. A slight shift to a lower wavenumber was observed as the RF power increased, regardless of the gas pressure. This could be indicative of a deviation from the stoichiometric level that changed with the RF power parameter [32]. At 3.5 Pa, the main peak was widened as the RF power increased, in agreement with the data extracted from the XRD spectra (see Figure 4). The sample deposited at 25 W showed the narrowest main band, supporting the improved crystalline quality. At 5.0 Pa, the narrowest band was obtained at 75 W, as the XRD data also revealed.

**Figure 7:**
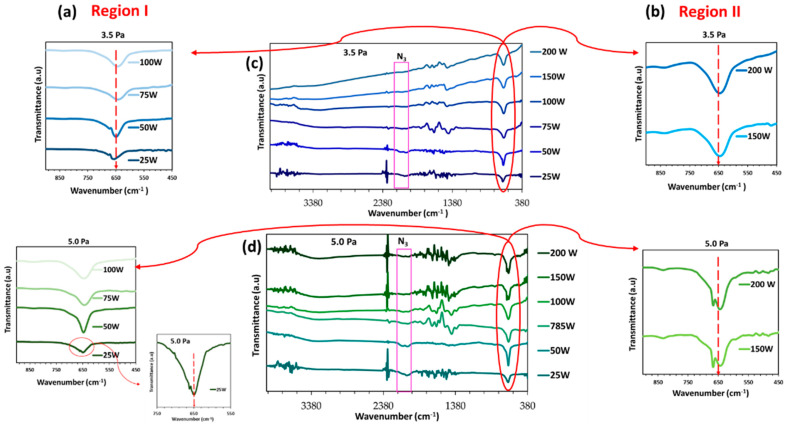
FTIR spectra of Cu_3_N films prepared at different RF power and gas pressures of 3.5 Pa and 5.0 Pa for samples in (**a**) region I and (**b**) region II. The whole spectra of the samples are pictured in (**c**,**d**).

Figure 7 depicts the FTIR spectra of the deposited Cu_3_N films. In all cases, a prominent peak around 650 cm^−1^ was observed, which was attributed to the intrinsic vibration of the Cu–N lattice mode [51]. The peak at ≈2049 cm^−1^ corresponded to the azide (N_3_) stretching vibration, confirming the formation of the Cu_3_N compound [52]. As the RF power increased, the intensity of the azide peak decreased, again related to the fact that, at a higher RF power, the Cu_3_N film became more Cu-rich, as previously observed from the EDS data (Table 4). In addition, an asymmetric peak with a shoulder at longer wavenumbers was obtained at lower RF power values (region I), which was more evident in the samples deposited at 3.5 Pa. Both the asymmetry and the shoulder began to disappear as both the RF power and the amount of gas were increased. This disappearance could be attributed to a better formation of the Cu-N bond due to the higher presence of Cu atoms as the RF power increased. The asymmetry and the shoulder also appeared in the samples of region II deposited at 5.0 Pa. This effect could be due to a possible imperfection in the formation of the Cu-N bond because of a greater presence within the plasma of both Cu atoms and energetic and ionized N species.

The resistivity of the films was measured with the four-point probe system. The results indicated a resistive material in all cases, where the values ranged from 0.2 × 10^3^ to 5.4 × 10^3^ Ω-cm. This film electrical performance was consistent with the chemical data and non-stoichiometric structure, richer in N than in Cu, derived from EDS and XRD measurements, respectively. This could be indicative of a p-type Cu_3_N material produced by the Cu^+^ vacancies due to the range of N_2_ gas pressure used. These values were similar to those found in the literature for Cu_3_N films deposited by the same technique [13,53,54,55].

**Figure 8:**
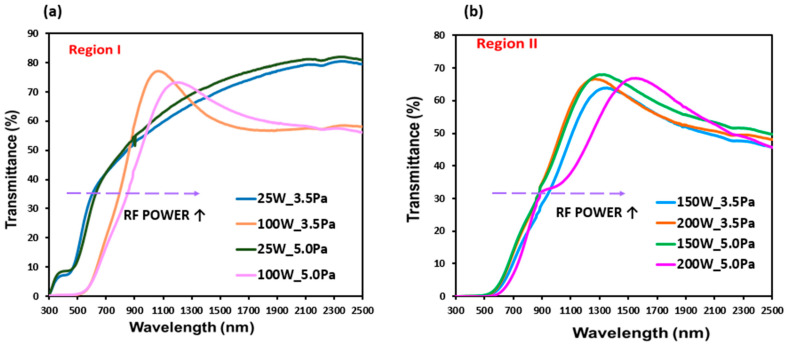
Transmittance spectra of Cu_3_N films prepared at different RF powers and working pressures. (**a**) region I; (**b**) region II.

Finally, the optical properties of the Cu_3_N thin films deposited on glass substrates were analyzed by UV-VIS-NIR spectroscopy in the range of 300–2500 nm. Figure 8 shows the transmittance spectra of the deposited films. In general, the films showed a high transmittance in the NIR (>700 nm), that gradually decreased in the VIS range (450–700 nm) and turned to very low values in the UV range (300–400 nm). It was also observed that, as the RF power increased, the average transmittance value was drastically affected, obtaining values ranging from 60% to 45%. This could be explained because the films were increasingly richer in Cu as the RF power rose, as previously reported [29,53]. A relationship between the increase in the thickness of the samples and the decrease in the percentage of transmittance was observed, which was attributed to the absorption of the material. In addition, the transmittance spectra showed a shift of the sharp absorption edge to lower energies as the RF power increased, as a result of the variation in the films composition and the structural change observed [56].

The optical band gap was calculated from the transmittance spectra using the Tauc Equation (3), which assumes the following energy-dependent absorption coefficient α [53]:

(3) $${\left(h\upsilon \alpha \right)}^{1/p}=A\left(h\nu -Eg\right)$$

where A is a constant, p is the parameter associated with the type of transition (i.e., p=2, for the indirect allowed transition and p=1/2, for the direct allowed transition), α is the absorption coefficient, and hν is photon energy. To calculate the band gap energy, we plotted the ${\left(h\nu \alpha \right)}^{1/2}$ and ${\left(h\nu \alpha \right)}^{2}$ vs. hν and extrapolated the straight-line portion of the curve with the x-axis to obtain the indirect and direct band gap, respectively. The results are summarised in Table 6.

**Figure 9:**
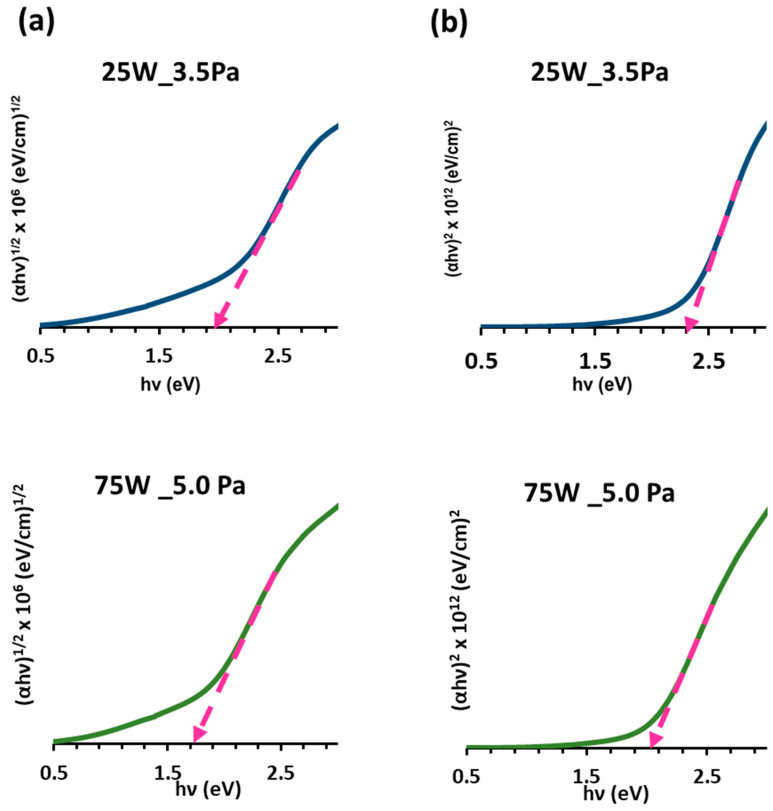
Plot of ${\left(h\nu \alpha \right)}^{1/2}$ and $\;{\left(h\nu \alpha \right)}^{2}$ vs. hν for the optimized samples deposited at (**a**) 25 W and 3.5 Pa, and (**b**) 75 W and 5.0 Pa.

The samples deposited at the lowest pressure of 3.5 Pa presented E_g_ values for the direct transitions ranging from 1.90 to 2.30 eV, and for the indirect allowed transitions, from 1.6 to 1.9 eV (Figure 9). In the case of the samples deposited at 5.0 Pa, the direct band gap energy ranged from 2.01 to 2.18 eV, and the indirect ranged from 1.70 to 1.80 eV. Therefore, a non-clear tendency with the gas pressure could be established and attributed to the different sample thicknesses. On the other hand, as RF power increased, a narrow band gap energy was obtained. This could be attributed to the increase in the amount of unbonded Cu atoms with RF power that formed electronic transitions from the defect levels to the valence band [57].

Finally, the values of the direct and indirect band gap obtained could be considered as suitable and within the range required to use this material as a solar absorber.

## 4. Conclusions

This work presented how the main properties of Cu_3_N thin films deposited by reactive magnetron sputtering can be tuned by changing the RF power and the gas pressure. All of the films were deposited on glass and silicon at RT in a pure N_2_ atmosphere.

Firstly, as the RF power increased, the deposition rate also rose, showing a linear trend but with two differentiated slopes depending on the power: 25–100 W (designated as region I) and 150–200 W (designated as region II). Such a division in regions was also observed in the rest of the film properties. From XRD, the films from region I showed a (100) preferred orientation, related to an N-rich material, that turned to the (111) preferred plane, related to a Cu-richer material, when the samples were deposited at an RF power of 150 W (region II). No difference was observed at the two different N_2_ pressures used. However, a better crystalline quality was obtained for the samples deposited at 25 W and 3.5 Pa, and at 75 W and 5.0 Pa. AFM measurements also revealed a different morphology in each case, changing from conical (region I) to nodular-like (region II) structures as the RF power increased. In addition, rougher surfaces were obtained for samples of region II, while the smoothest surfaces were achieved at 5.0 Pa. Regarding the chemical composition, qualitative EDS measurements indicated a gradual and slight increase in Cu as the RF power increased. FTIR and Raman confirmed the formation of Cu-N bonds, with a slight displacement to a lower wavenumber as the RF power increased. From the electrical point of view, all of the films showed high resistivity. The band gap energy obtained ranged from 1.60 to 1.90 eV for the indirect transitions, and 1.90 to 2.30 eV for the direct transitions; suitable ranges for the proposed application. In summary, this work demonstrated: (i) the feasibility of magnetron sputtering to manufacture a stable Cu_3_N material at RT; (ii) how the material properties strongly depend on deposition parameters; and (iii) which sputtering parameters lead to a material useful for use as a solar absorber (i.e., good structural quality, smooth surfaces, large grain size, stable Cu-N bond and adequate bandgap value). We could conclude that the samples prepared at a N_2_ pressure of 5.0 Pa and RF power values between 50 and 100 W would be desirable candidates as solar absorbers. Lastly, the specific solar cell technology that could benefit from the use of this film material could be thin-film solar devices, as they provide a flexible texture, and are cheap to manufacture. 

## Figures and Tables

**Table 1:** Sputtering deposition conditions used to fabricate the Cu_3_N films at RT in N_2_ pure atmosphere at different RF powers and gas pressures. Film thickness is also included.

**Total Gas Pressure: 3.5 Pa**						
RF Power (W)	25	50	75	100	150	200
Film thickness (nm)	45	85	95	100	175	215
Deposition time (s)	1800	1800	900	900	600	420
**Total Gas Pressure: 5.0 Pa**						
RF Power (W)	25	50	75	100	150	200
Film thickness (nm)	40	85	90	100	155	210
Deposition time (s)	1800	1800	900	900	600	420

**Table 2:** Main XRD data extracted from the XRD spectra of the Cu_3_N films fabricated on glass by reactive RF magnetron sputtering in pure N_2_ atmosphere.

***Total Gas Pressure: 3.5 Pa***						
RF Power (W)	25	50	75	100	150	200
2θ (°)	23.327	23.248	23.203	23.272	40.746	40.748
Predominant direction	(100)	(100)	(100)	(100)	(111)	(111)
Lattice parameter a (nm)	0.3810	0.3826	0.3833	0.3822	0.3836	0.3833
FWHM (*°*)	0.048	0.138	0.1771	0.1181	0.1771	0.1771
Grain size (nm)	30	31	27	36	30	32
***Total Gas Pressure: 5.0 Pa***						
RF Power (W)	25	50	75	100	150	200
2θ (°)	23.297	23.214	23.343	23.251	40.794	23.293
Predominant direction	(100)	(100)	(100)	(100)	(111)	(100)
Lattice parameter a (nm)	0.3818	0.3832	0.3817	0.3826	0.3840	0.3814
FWHM (°)	0.1378	0.0787	0.048	0.2362	0.0787	0.6298
Grain size (nm)	27	36	33	38	27	33

**Table 3:** Surface roughness RMS and grain size calculated from AFM 1 × 1 µm^2^ images of the Cu_3_N films fabricated on glass by RF magnetron sputtering in a pure nitrogen atmosphere.

**Total Gas Pressure: 3.5 Pa**						
RF Power (W)	25	50	75	100	150	200
RMS (nm)	8.50	2.20	3.25	1.77	8.81	7.40
Grain size (nm)	31	31	33	34	36	37
**Total Gas Pressure: 5.0 Pa**						
RF Power (W)	25	50	75	100	150	200
RMS (nm)	1.50	1.33	1.50	8.02	5.60	5.70
Grain size (nm)	29	35	33	34	32	33

**Table 4:** Relative chemical composition of the Cu_3_N films in the study, derived from EDS analysis.

RF Power (W)	25	50	75	100	200
Cu/N ratio (3.5 Pa)	1.85	1.94	1.70	2.15	2.33
Cu/N ratio (5.0 Pa)	1.87	2.06	2.36	2.48	2.87

**Table 5:** Main parameters of Cu_3_N films RF power derived from the Raman spectra.

RF Power (W)	25	50	75	100	150	200
FWHM (cm^−1^) (3.5 Pa)	66	82	105	99	121	114
Raman peak (cm^−1^)	632	637	621	621	616	616
FWHM (cm^−1^) (5.0 Pa)	108	120	107	118	108	77
Raman peak (cm^−1^)	644	640	627	620	616	616

**Table 6:** Direct and indirect band gap energy values calculated for the Cu_3_N films fabricated on glass in a pure N_2_ atmosphere at different RF powers.

**Total Gas Pressure: 3.5 Pa**						
RF Power (W)	25	50	75	100	150	200
Eg (eV)						
Direct (αhν)^2^	2.30	2.10	2.05	2.05	2.00	1.90
Indirect (αhν)^1/2^	1.90	1.80	1.70	1.70	1.60	1.70
**Total Gas Pressure: 5.0 Pa**						
RF Power (W)	25	50	75	100	150	200
Eg (eV)						
Direct (αhν)^2^	2.10	2.18	2.05	2.01	2.05	2.10
Indirect (αhν)^1/2^	1.80	1.80	1.70	1.70	1.70	1.70

## Data Availability

Not applicable.
